# Dysregulation of Circadian Rhythm Gene Expression in Cystic Fibrosis Mice

**DOI:** 10.5334/jcr.175

**Published:** 2019-04-18

**Authors:** Eric Barbato, Hannah Mianzo, Paul Litman, Rebecca Darrah

**Affiliations:** 1 Case Western Reserve University, US; 2 Cincinnati Children’s Hospital Medical Center, US

**Keywords:** Cystic Fibrosis, circadian rhythm, gene expression

## Abstract

Cystic fibrosis (CF) is autosomal recessive disease that affects multiple body systems. CF patients often experience sleep disturbances, altered sleep patterns, and sleep apnea. Sleep in mammals is controlled in part by circadian clock genes, including *Clock, Bmal1, Period1, Period2, Cryptochrome1,* and *Cryptochrome2*. The purpose of this study was to gain a better understanding of the biological underpinnings of disordered sleep experienced in CF. To accomplish this, we evaluated circadian clock gene expression profiles in CF and wildtype mice, divided into two subgroups each based on sleep condition. One subgroup of each genotype was permitted to maintain their sleep-wake cycle while the other was deprived of sleep for six hours prior to sacrifice. Brain, skeletal muscle, jejunum, colon, lung and adipose tissues were collected from each mouse. Quantitative polymerase chain reaction (PCR) was used to quantify expression of *Clock, Bmal1, Period1, Period2, Cryptochrome1* and *Cryptochrome2,* and expression levels were compared between study groups. Our comparisons showed distinct differences between the CF groups and the wildtype groups under both sleep conditions. Additionally, we found the CF mice that had been sleep deprived had severely dysregulated expression of all measured genes in the lung apart from *Cry1*. Our findings suggest that (1) disordered sleep in CF may be caused by circadian system dysregulation and (2) the loss of the cystic fibrosis transmembrane conductance regulator (CFTR) is a causative factor in the dysregulated circadian clock gene expression profiles of CF mice.

## Introduction

Cystic Fibrosis (CF), an autosomal recessive disease that affects approximately 1 in 2,500 Caucasian newborns, is caused by mutations in the *cystic fibrosis transmembrane conductance regulator* (*CFTR*) gene. Though the disease affects nearly every body system, the respiratory and digestive systems are the primary sources of morbidity and mortality in CF [1]. Due to their disproportionately high contribution to complications in CF, these systems are often the most heavily studied. CF patients, however, experience many other clinically relevant symptoms such as poor sleep quality [2,3]. Previous research in the field has suggested that children and adults with CF commonly experience sleep disturbances, altered sleep patterns and sleep apnea, all of which contribute to poor sleep quality [3,4,5,6].

Poor sleep quality can contribute to many health concerns in otherwise healthy individuals, let alone those with life-limiting diseases such as CF. Poor sleep quality has been associated with alterations in metabolic profiles, which can cause insulin resistance and dysregulated sympathetic nervous activity [7]. Of particular concern to CF patients, poor sleep quality has also been associated with impaired immune response and increased susceptibility to infectious diseases [8]. Furthermore, poor sleep quality can increase the risk of depression and anxiety [9]. Cognitive performance has also been shown to decline with consecutive hours of sleep deprivation, specifically in relation to complex task performance such as decision making, multitasking, memory tasks, and team performance tasks [10].

While it is documented that patients with CF experience lower sleep quality, the underlying pathophysiologic mechanisms responsible have yet to be fully characterized. It has long been a clinical assumption that CF-related respiratory symptoms were the primary cause of the widespread sleep disturbances in CF. Indeed, most research on the topic has supported this notion, hypothesizing the cause to be coughing and/or hypoxemia in addition to CF medication side effects and disease-related anxiety [6,11]. While these hypotheses are logical, some evidence suggests that these factors may not fully explain the poor sleep quality associated with CF. A 2012 report indicated that children with CF experience similar nocturnal respiratory profiles to those of healthy children [12]. This similarity suggests that there may be other factors causing poor sleep quality in individuals with CF. Furthermore, recent research has shown that sleep disturbances experienced by individuals with CF are consistent with circadian system phase delays [13,14], implicating the circadian system as a possible culprit in the disordered sleep of CF patients.

Sleep onset and duration are mediated by the circadian rhythms, a set of cyclic mechanisms that act as a mammal’s internal body clock. This internal regulation of physiological and behavioral processes allows for the body to anticipate and react to changes in the environment, such as sunlight [15,16]. A specific set of genes is responsible for the control of the circadian system including *Clock, Bmal1, Period1, Period2, Cryptochrome1*, and *Cryptochrome2*, all of which encode proteins of the same names. *Clock* and *Bmal1* are components of a positive feedback loop, which dimerize with each other and subsequently activate the transcription of *Period* and *Cryptochrome. Period* and *Cryptochrome* then dimerize and act on the *Clock/Bmal1* heterodimer to repress the transcription of *Period* and *Cryptochrome* [17]. The tissues that have been found to express *Clock, Bmal1, Period1 (Per1), Period2 (Per2), Cryptochrome1 (Cry1),* and *Cryptochrome2 (Cry2)* include white blood cells, lymph nodes, brain, heart, skeletal muscle, colon, adipocytes, kidney, liver, lung, thyroid, adrenal gland, breast, ovary, prostate, and testis.

Given the implication of the circadian system in the disordered sleep experienced by individuals with CF, we hypothesized that expression of the circadian clock genes would be dysregulated in CF. To test our hypothesis, we examined expression of the circadian clock genes in multiple tissues of both CF and wildtype (WT) mice under two sleep conditions.

## Materials and methods

### Mouse models and experimental groups

C57BL/6 wild type mice and CF mice homozygous for the F508del CFTR mutation were used in this study. These mice are a commonly used model for the F508del CFTR mutation in humans and recapitulate most of the human phenotype associated with F508del homozygosity [18]. Mice were age-matched at an average 7.1 weeks of age at the time of sacrifice. Twenty-three mice in total, consisting of both genders (9 female and 14 male), were equally divided into 4 categories. Control mice (WT and CF “normal sleep”) were allowed to sleep undisturbed, with no external interference until immediately before sacrifice. Experimental groups (WT and CF “sleep deprived”) were kept awake for 6 hours using constant, gentle physical agitation/manipulation, starting at 8am immediately following their nocturnal active phase [19,20,21]. Because of the nocturnal nature of mice and the fluctuation of circadian clock gene expression throughout the 24-hour cycle, the above protocols were completed during the day and all mice were sacrificed at the same time [15,17,21]. Animal breeding, housing, sacrifice and tissue collection were approved by the Institutional Animal Care and Use Committee of Case Western Reserve University.

### Tissue collection and RNA extraction

Mice were euthanized with exposure to CO2 and sections of brain (suprachiasmatic nucleus (SCN) and immediately surrounding tissue), skeletal muscle, jejunum, colon, adipose tissue and lung were collected and flash frozen in liquid nitrogen. Total RNA was phenol extracted from an ~2mm diameter section of each tissue following mechanical homogenization in Ribosol (Amresco). RNA was then precipitated in isopropanol, washed twice with 75% and 95% EtOH, and resuspended in 200uL nuclease-free ddH20. An additional on-column DNase I treatment for 20 minutes at 23°C and RNAeasy spin-cup purification (Qiagen) following the manufacturer’s protocol was required to remove residual DNA carryover. RNA concentration was quantified with a Nanodrop spectrophotometer, and 1ug of total RNA was converted to cDNA at 42° for 30 minutes using a qScript cDNA synthesis kit as per manufacturer’s protocol (Quantabio).

### Gene expression and analyses

Quantitative Real-Time PCR using standard TaqMan assays (Thermo Life Technologies) was performed to determine relative gene expression of *Clock* (Mm00455950_m1), *Per1* (Mm00501813_m1), *Per2* (Mm00478113_m1), *Bmal1* (Mm00500226_m1), *Cry1* (Mm00514392_m1), and *Cry2* (Mm01331539_m1), and were multiplexed with β-actin as an endogenous control. All reactions were run for 50 cycles at 60°C following a 95°C denaturation step. cDNA controls containing no reverse-transcriptase were also run at 50 cycles on each RNA isolate to verify that no identifiable genomic carryover was present in the template. Cycle thresholds (Ct) were determined using StepOnePlus software. The mean cycle threshold difference (ΔCt) between the genes of interest and the endogenous controls was calculated for each group (WT normal sleep, WT sleep deprived, CF normal sleep, and CF sleep deprived) and ΔΔCt for each group was calculated relative to WT normal sleep as a baseline. Fold difference (2^–ΔΔCt^) is reported as a percent of the WT normal sleep baseline.

Independent-samples t-tests were calculated to compare the circadian clock gene profiles of 1.) Rested WT mice vs. Rested CF mice 2.) Sleep deprived WT mice vs. sleep deprived CF mice 3.) Rested WT mice vs. sleep deprived WT mice 4.) Rested CF mice vs. sleep deprived CF mice. After using the Bonferroni correction for multiple comparisons, statistical significance was considered p < 0.008.

## Results

We organized our experiments into the following four group comparisons:

1. WT rested vs. CF rested
2. WT sleep deprived vs. CF sleep deprived
3. WT rested vs. WT sleep deprived
4. CF rested vs. CF sleep deprived

Within each group, we measured the expression of the following genes: *Clock, Bmal1, Per1, Per2, Cry1,* and *Cry2* in the following tissues: brain, jejunum, fat, colon, lung, and skeletal muscle.

### Group comparison 1: WT rested vs. CF rested

To determine whether the circadian clock gene expression profiles of CF mice differ from those of WT mice at baseline, we compared gene expression data from our WT mice and CF mice that had all been allowed to sleep normally. We found that in the rested state, CF mice had increased expression of *Clock* in the brain (t_6.09_ = 7.48, p < .001) and jejunum (t_7.94_ = 4.09, p = .008), increased expression of *Bmal1* in the jejunum (t_8.91_ = 3.8, p = .004), and increased expression of *Cry2* in the brain (t_9_ = 7.03, p < .001). All other comparisons between these two groups produced no statistically significant results (Table 1).

**Table 1:** P values of independent-samples T-Tests comparing average ΔΔCt of the CF rested group to the WT rested group. Statistically significant (p ≤ .008) increases are highlighted in dark gray.

Tissue Type	*Clock*	*Bmal1*	*Per1*	*Per2*	*Cry1*	*Cry2*

**Brain**	<.001	.568	.467	.795	.165	<.001
**Jejunum**	.008	.004	.108	.419	.072	.233
**Adipose**	.232	.314	.712	.969	.214	.169
**Colon**	.936	.054	.603	.245	.669	.363
**Lung**	.642	.824	.804	.915	.933	.626
**Skeletal Muscle**	.294	.681	.282	.405	.101	.07

**Figure 1:**
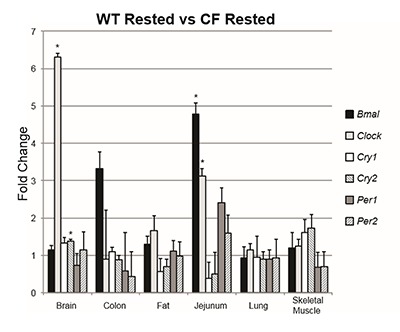
Fold change of the WT rested group vs. the CF rested group in each measured transcript grouped by tissue. Bars represent gene expression of the CF rested group, whereas the WT rested group is represented by 1 on the Y axis. Asterisks indicate statistical significance (p ≤ 0.008).

To more clearly visualize gene expression differences between the WT rested and CF rested groups, we also calculated fold change of the CF group vs the WT group (Figure 1).

### Group comparison 2: WT sleep-deprived vs. CF sleep deprived

**Figure 2:**
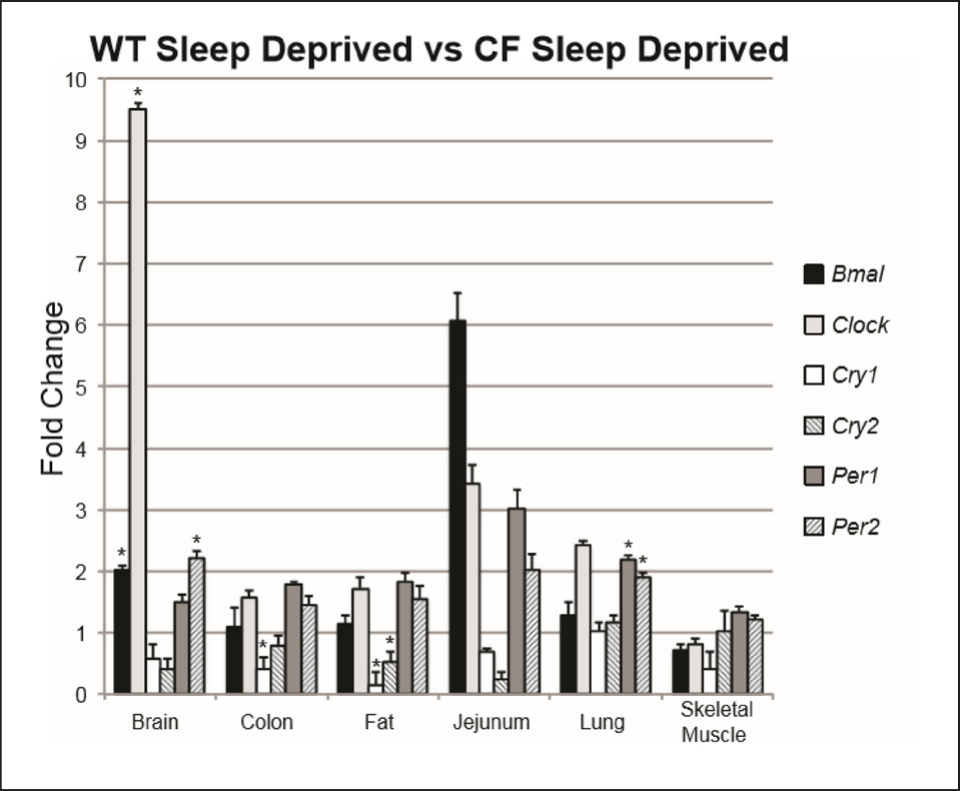
Fold change of the WT sleep deprived group vs. the CF sleep deprived group in each measured transcript grouped by tissue. Bars represent gene expression of the CF sleep deprived group, whereas the WT sleep deprived group is represented by 1 on the Y axis. Asterisks indicate statistical significance (p ≤ 0.008).

To evaluate the effect of sleep deprivation on WT and CF mice, we compared the two groups after both had been sleep deprived for six hours prior to analysis. We found that, in the sleep deprived state, CF mice had significantly dysregulated expression of circadian clock genes compared to WT mice. The CF group showed increased expression of *Clock* (t_7.21_ = 13.42, p < .001) and *Bmal1* (t_8.84_ = 5.46, p < .001) in the brain. *Per1* expression in the lung was increased in the CF group compared to WT (t_10_ = 3.56, p = .005). *Per2* was also dysregulated; the CF group showing increased expression in the brain (t_9.53_ = 5.47, p < .001) and lung (t_8.3_ = 4.95, p = .001). Finally, *Cry1* (t_10_ = –10.2, p < .001) and *Cry2* (t_7.09_ = –9.55, p < .001) expression were decreased in the adipose tissue as well as *Cry1* in the colon (t_10_ = –3.42, p = .007) (Table 2, Figure 2).

**Table 2:** P values of independent-samples T-Tests comparing average ΔΔCt of the WT sleep deprived group to the CF sleep deprived group. Statistically significant (p ≤ .008) increases are highlighted in dark gray and decreases in light gray. Asterisks indicate statistical significance (p ≤ 0.008).

Tissue Type	*Clock*	*Bmal1*	*Per1*	*Per2*	*Cry1*	*Cry2*

**Brain**	<.001	<.001	.046	<.001	.036	.193
**Jejunum**	.018	.010	.017	.048	.057	.306
**Adipose**	.067	.611	.025	.081	<.001	<.001
**Colon**	.208	.848	.101	.171	.007	.065
**Lung**	.023	.372	.005	.001	.911	.918
**Skeletal Muscle**	.137	.069	.126	.204	.016	.077

### Group comparison 3: WT rested vs. WT sleep deprived

**Figure 3:**
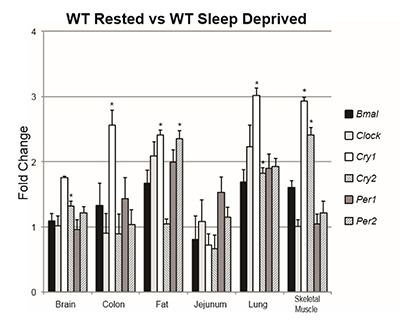
Fold change of the WT rested group vs. the WT sleep deprived group in each measured transcript grouped by tissue. Bars represent gene expression of the WT sleep deprived group, whereas the WT rested group is represented by 1 on the Y axis. Asterisks indicate statistical significance (p ≤ 0.008).

To determine the effect of sleep deprivation in the absence of CF, we compared the WT group that had slept normally to the WT group that had been sleep deprived for six hours prior to analysis. We found that when compared to the rested group, the sleep deprived group had increased expression of *Per2* in adipose tissue (t_8.98_ = 4.26, p = .002). The sleep deprived group had significantly dysregulated expression of *Cry1* in the adipose tissue (t_5.45_ = 2.78, p = .005), colon (t_10_ = 3.58, p = .005), lung (t_6.26_ = 3.76, p = .004), and skeletal muscle (t_10_ = 12.05, p < .001). *Cry2* was also severely dysregulated between these groups, with the sleep deprived group showing increases in the brain (t_10_ = 3.54, p = .005), lung (t_7.47_ = 3.81, p = .006), and skeletal muscle (t_9.68_ = 7.04, p < .001) (Table 3, Figure 3).

**Table 3:** P values of independent-samples T-Tests comparing average ΔΔCt of the WT rested group to the WT sleep deprived group. Statistically significant (p ≤ .008) increases are highlighted in dark gray.

Tissue Type	*Clock*	*Bmal1*	*Per1*	*Per2*	*Cry1*	*Cry2*

**Brain**	.962	.717	.855	.487	.011	.005
**Jejunum**	.860	.653	.323	.716	.381	.285
**Adipose**	.021	.047	.016	.002	.005	.822
**Colon**	.831	.581	.454	.916	.005	.693
**Lung**	.072	.075	.129	.111	.004	.006
**Skeletal Muscle**	.960	.067	.798	.443	<.001	<.001

### Group comparison 4: CF rested vs. CF sleep deprived

**Figure 4:**
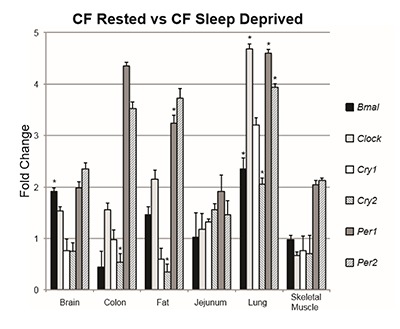
Fold change of the CF rested group vs. the CF sleep deprived group in each measured transcript grouped by tissue. Bars represent gene expression of the CF sleep deprived group, whereas the CF rested group is represented by 1 on the Y axis. Asterisks indicate statistical significance (p ≤ 0.008).

Finally, to more fully understand the role of sleep deprivation in the presence of CF, we compared CF mice that had slept normally to CF mice that had been sleep deprived for six hours prior to analysis. Unlike the WT mice, the sleep deprived CF mice showed increased expression of *Bmal1* in the brain (t_6.08_ = 5.32, p = .002) and increased expression of *Per1* in the adipose tissue (t_6.47_ = 4.09, p = .005). Of note, comparison between these groups revealed increases in expression in the lung of all measured gene transcripts apart from *Cry1* also not seen in the WT mice: *Clock* (t_6.14_ = 8.86, p < .001), *Bmal1*: (t_7.86_ = 2.57, p = .003), *Per1*: (t_9_ = 7.3, p < .001), *Per2*: (t_9_ = 3.39, p = .008), *Cry2*: (t_9_ = 4, p = .003). Finally, *Cry2* expression was decreased in the adipose tissue (t_9_ = –5.17, p = .001) and colon (t_9_ = –3.45, p = .007), which was also dissimilar to the WT comparison (Table 4, Figure 4).

**Table 4:** P values of independent-samples T-Tests comparing average ΔΔCt of the CF rested group to the CF sleep deprived group. Statistically significant (p ≤ .008) increases are highlighted in dark gray and decreases in light gray.

Tissue Type	*Clock*	*Bmal1*	*Per1*	*Per2*	*Cry1*	*Cry2*

**Brain**	.010	.002	.077	.041	.285	.112
**Jejunum**	.632	.965	.202	.473	.409	.28
**Adipose**	.095	.162	.005	.014	.167	.001
**Colon**	.678	.125	.107	.103	.908	.007
**Lung**	<.001	.003	<.001	.008	.056	.003
**Skeletal Muscle**	.076	.934	.064	.036	.472	.431

## Discussion

Here we report that CF mice allowed to sleep normally exhibit significant increases in the expression of *Clock* and *Cry2* in the brain and *Clock* and *Bmal1* in the jejunum compared to their WT littermates. However, given the data regarding altered sleep profiles of CF patients, we also compared CF mice to WT mice when both groups had been deprived of sleep for six hours. In this comparison, we found similar alterations in the expression of *Clock* in the brain, though the change in *Cry2* was not present. In addition, we found that the expression of *Bmal1* and *Per2* was also increased in the brain, though there were no significant changes in the jejunum, and *Per1* and *Per2* were increased in the lung. We also found significant decreases in *Cry1 and Cry2* in the adipose tissue and *Cry1* in the lung. Given there was only a minor overlap in these two comparisons, these data suggest that the loss of CFTR may cause dysregulation in the expression of some circadian clock genes which is exacerbated by sleep deprivation. Additionally, while it is known that there are positive and negative feedback mechanisms present in circadian system control, the changes in relative gene expression we detected in peripheral tissues did not correlate with changes in the SCN. These data provide additional evidence that the dysregulation we detected may be due to the loss of CFTR rather than abnormal gene expression in the SCN.

While we did not measure circadian behaviors, our previously published data indicate that CF mice homozygous for F508del display increased energy expenditure during normal sleep hours when compared to their WT littermates [22]. These experiments also did not measure activity or locomotion but taken together with the changes in circadian clock gene expression between CF and WT mice, these data indicate that differences between these genotypes may extend beyond clock gene expression to affect circadian behaviors as well. Further investigation of circadian behaviors is required to more fully explore the apparent relationship between circadian clock gene regulation and behavior in CF and WT mice.

In order to determine whether these changes could be attributed to sleep deprivation alone, we compared the WT rested group to the WT sleep deprived group. In this comparison, we found increases of *Per2* expression in the adipose tissue. Interestingly, *Cry1* and *Cry2* were also dysregulated, but in a different manner than before. When compared to the WT rested group, the WT sleep deprived group had increases of *Cry1* expression in the adipose tissue, lung, colon, and skeletal muscle. *Cry2* expression was also increased in the brain, lung, and skeletal muscle. Taken together, these data indicate that the WT mice experience alterations in circadian clock gene expression when sleep deprived, but in a manner not consistent with that of the CF mice.

Finally, to evaluate the effect of sleep deprivation in the absence of functional CFTR, we compared the CF rested group to the CF sleep deprived group.

We found that when compared to the CF rested group, the CF sleep deprived group also had a significantly dysregulated gene expression profile, but again, in a manner not consistent with their WT counterparts. This comparison revealed that the sleep deprived group had increases of *Bmal1* expression in the brain and *Per1* in the adipose tissue. Additionally, we found decreased expression of *Cry2* in the adipose tissue and colon whereas it was unchanged in the WT sleep deprived group compared to the WT rested group.

Perhaps our most interesting finding was the increase of every measured transcript apart from *Cry1* in the CF sleep deprived lung. Though CF is a multi-organ system disease, the lung contributes disproportionately greater to CF-related morbidity and mortality, due primarily to infection, inflammation, and declining lung function. Previous reports have implicated alteration in circadian clock gene expression as a profound modulator of lung function as a whole [23,24,25,26]. Given that CF mice have been shown to demonstrate altered pulmonary mechanics in the absence of intentional sleep deprivation, our present findings may be a potential explanation for these changes in pulmonary phenotype [27].

With regard to lung infection, circadian clock genes have previously been implicated as regulators of the immune inflammatory response in the lung [28]. Interestingly, other reports have also suggested that circadian clock gene expression may be altered in response to bacterial lung infection [29,30]. Furthermore, CF mice have been shown to exhibit impaired immune response [31,32]. However, given that the mice used in our experiments were free of detectable lung infection, the altered circadian clock gene expression profiles we observed in the CF sleep deprived group may be related the dampened immune capacity of CF mice.

In summary, there was a distinct difference between the WT groups and CF groups under both sleep conditions. The data presented here show that both lack of CFTR and sleep deprivation are associated with significant and varied alterations in the circadian clock gene expression of C57BL/6 mice. Furthermore, the difference in transcription between the presence of CF and sleep deprivation suggests that the loss of function of CFTR may be a causative factor in the alterations of the gene expression profiles we observed. Lastly, coupled with previously published data on lung mechanics and infection in CF, our findings in the lung tissue of CF mice indicate that alterations in lung-based expression of circadian clock genes may contribute to changes in pulmonary mechanics and infection.

In addition, while our observations were made in pre-clinical models, they are consistent with and extend previous reports suggesting that (1) disordered sleep in CF may be related to circadian system dysregulation and (2) disordered sleep may be a primary effect of the loss of CFTR as opposed to a consequence of CF-related symptoms [13].

## Limitations and Future Directions

Limitations of our design include limited sample size, variance in age of the mice, limited diversity of CFTR genotype, and measurement of circadian clock genes at only a single time point. Given that our experimental groups included only 5–6 mice each, our data were particularly susceptible to variance within each group affecting group averages. Furthermore, after correcting for multiple comparisons, our threshold for statistical significance was .008. Since many of our comparisons yielded results that were statistically significant at a slightly more liberal p value, higher sample sizes may provide enough power to detect those associations. Furthermore, with our limited sample sizes, we were unable to investigate differences between male and female mice. A 2012 report indicated that expression of *Clock* and *Bmal1* varied between male and female mice under the same experimental conditions [24], therefore further investigation of sex-based differences in circadian clock gene expression is warranted.

Though all experimental mice were adults, the ages of mice used in this study ranged from 5.2 weeks to 9.4 weeks, with an average of 7.1 weeks. The mice were divided into two groups based on genotype, then randomly assigned to a subgroup (rested or sleep deprived). Since we did not take age into account when assigning groups, it remains unclear if age had any effect on the gene expression profiles of our mice.

We studied only WT C57BL/6 mice and CF mice homozygous for F508del, also on a congenic C57BL/6 background. Given that F508del accounts for over 70% of mutated alleles in CF patients and F580del homozygosity produces a particularly severe phenotype in mice, it was logical to begin testing our hypothesis using this genotype. However, different CF-causing mutations produce different phenotypes in both CF patients and CF mice. Thus, further study is required to more fully understand the role of circadian clock gene expression in CF as a whole by examining the gene expression profiles of CF mice with diverse CFTR genotypes.

It is known that natural fluctuation in the transcription of circadian clock genes over time influences different aspects of the circadian system. Though our experiments revealed interesting differences in circadian clock gene expression between genotypes and sleep conditions at a single time point, further measurement of transcription at different time points is required to more fully understand circadian system oscillation.
